# SYBERT’S KERATODERMA IN THREE SIBLINGS

**DOI:** 10.4103/0019-5154.70683

**Published:** 2010

**Authors:** Varadraj V Pai, Sanath Rao, K N Naveen

**Affiliations:** *From the Department of Dermatology, SDM College of Medical Sciences and Hospital, Dharwad, India. E-mail: docpai@rediffmail.com*

Sir,

In 1988, Sybert described a rare form of transgradient keratoderma which resembled mal-de-melada but had autosomal dominant inheritance.[1] In this complex diffuse keratoderma, the skin lesions are characterized by palmoplantar hyperkeratosis with transgradiens and progradiens, nail and dental abnormalities, and the absence of systemic manifestations.

In a family of five siblings, two sisters aged 14 and 12 and their brother aged 4 years presented to the OPD with a history of thickening and scaling over the palms and soles since their birth. History of progression with deformity and a similar family history for the mother. There was no history of consanguinity and the other two siblings were normal.

**Figure 1:**
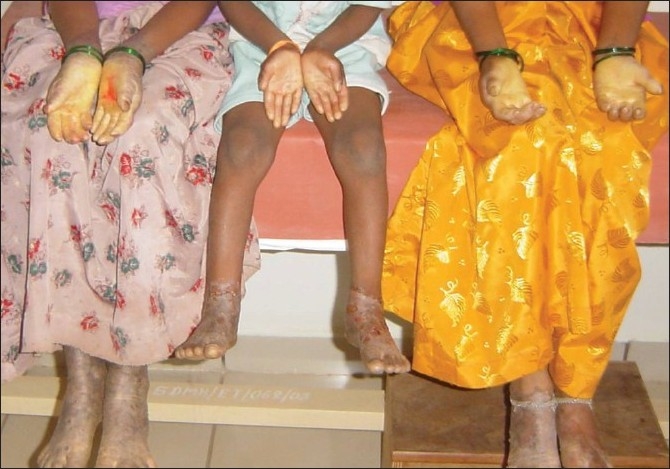
Diffuse hyperkeratosis of the palms and soles extending on to the dorsum of the hand and feet

**Figure 2:**
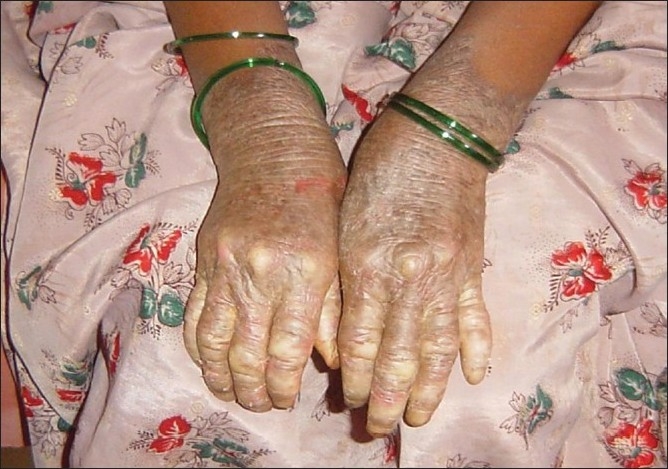
Pseudoainhum formation of the little finger

Examination revealed the presence of hyperkeratosis of the palms and soles which extended on to the dorsum of the hands and the feet [Figure 1]. There was pseudoainhum formation of the little fingers of the older two siblings [Figure 2] and onychodystrophy of the nails of the fingers and toes. Systemic examination did not reveal any abnormalities and biopsy was not done due to the lack of consent from the patients’ attender.

A diagnosis of Sybert’s keratoderma was made based on the clinical findings and the patients were treated with topical keratolytics and moisturizers. Clinical improvement was seen with respect to the extent of palmar hyperkeratosis on follow-up after four weeks.

Hereditary palmoplantar keratodermas (PPK) are a heterogeneous group of diseases characterized by hyperkeratosis of the palms and soles with a thickening of the stratum corneum, usually distinguishable by the mode of inheritance and by associated clinical findings.

The inheritance may be autosomal dominance, autosomal recessive, or X-linked.[1]

Palmoplantar keratodermas has been historically based on the spread to the wrist, dorsa of the hand, knuckles, elbow, and knee (transgradiens), and progression with age (progradiens).[1]

Clinically, three distinct patterns of palmoplantar keratodermas are seen:[2]

1. Diffuse palmoplantar keratodermas characterized by even, thick, symmetrical hyperkeratosis all over the palms or soles.
2. Focal palmoplantar keratodermas in which large compact masses of keratin develop at the site of recent friction.
3. Punctuate palmoplantar keratodermas in which multiple, tiny, ‘raindrop’ keratoses involve the palmoplantar surface.

Keratodermas are additionally classified into three subgroups:[3]

- Simple keratodermas with only palmoplantar keratodermas
- Complex keratodermas: palmoplantar keratodermas with lesions on nonvolar skin, hair, teeth, nails, and sweat glands.
- Syndromic keratodermas: palmoplantar keratodermas associated with abnormalities of other organs including deafness and cancer.

Based on the above classification, all three patients were diagnosed with a diffuse complex palmoplantar keratoderma with transgradiens and progradiens and an autosomal dominant inheritance. The differential diagnosis to this subset of palmoplantar keratodermas was a milder Grither’s syndrome and a severe Sybert’s keratoderma. Sybert’s keratoderma differs from Grither’s keratoderma with respect to its early onset, progression, and greater severity characterised by pseudoainhum formation as well as glove and stocking hyperkeratosis, which were present in our patients.[4] Histopathologically, Sybert’s keratoderma shows the presence of lipid-laden cells in the stratum corneum. Good clinical response is seen with systemic isotretinoin.[5] we report this case because of its rarity.
